# Fortification of Sunflower Oil by Nanoemulsions Containing Vitamin‐D_3_ : Formation, Stability, and Release

**DOI:** 10.1002/fsn3.4677

**Published:** 2025-03-14

**Authors:** Nadia Ahamdi, Parham Joolaei Ahranjani, Ladan Rashidi, Keramatollah Rezaei

**Affiliations:** ^1^ Food Technology and Agriculture Products Research Center Standard Research Institute (SRI), Iranian National Standards Organization (INSO) Karaj Iran; ^2^ Faculty of Agricultural, Environmental and Food Sciences Free University of Bolzano Bolzano Italy; ^3^ Department of Food Science and Engineering, Faculty of Agricultural Engineering and Technology University of Tehran Karaj Iran

**Keywords:** food‐grade encapsulation, nanoemulsions, sunflower oil fortification, vitamin‐D_3_, whey protein concentration (WPC)

## Abstract

This study addresses the challenge of stabilizing vitamin D_3_, an unstable, fat‐soluble vitamin, whose efficacy is diminished by environmental factors. The objective was to encapsulate vitamin D_3_ using pectin (1%–3% w/w) and whey protein concentrate (WPC) (1%–2% w/w) at varying ratios, facilitated by Tween 80 surfactant (0.5% and 2.5% w/w), through high‐pressure homogenization to create oil‐in‐water (O/W) nanoemulsions. Optimization of the preparation conditions for both aqueous and oil phases was conducted using an experimental design. Characterization and stability of the nanoemulsions were assessed using scanning electron microscopy (SEM), dynamic light scattering (DLS), and Fourier transform infrared spectroscopy (FTIR). Release kinetics of vitamin‐D_3_ into sunflower oil were monitored using high‐performance liquid chromatography (HPLC) under various conditions. The optimal encapsulation was achieved with a 30:70 oil‐to‐aqueous phase ratio, comprising 27.5% oil and 2.5% surfactant in the oil phase, and 1% WPC and 2% pectin in the aqueous phase. The nanoemulsion demonstrated stability over 60 days of storage, with a *z*‐average particle size of 98.2 nm. HPLC analysis indicated a 90% recovery of encapsulated vitamin‐D_3_ in sunflower oil. These findings suggest the promising approach of the developed nanoemulsion for enhancing the bioavailability and shelf life of vitamin‐D_3_ in food applications.

## Graphical abstract

Vitamin D_3_ (VIT D) was encapsulated in oil‐in‐water (Q/W) nanoemulsion. Optimal encapsulation was obtained at 27.5% oil, 2.5% surfactant, 1% WPC, and 2% pectin. The nanoemulsion stability was over 60 days of storage with a *z*‐average particle size of 98.2 nm. HPLC analysis indicated a 90% recovery of encapsulated VIT D in sunflower oil.

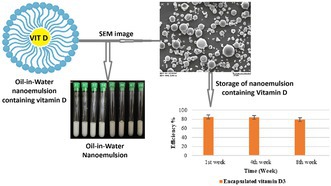

## 1 Introduction

Nanoemulsions, as a subclass of colloidal dispersions, are characterized by their fine particle size, typically ranging from 20 to 200 nm, dispersed within an aqueous medium (Rolland et al. 2021). These systems have garnered significant interest in the food industry due to the unique physicochemical and biological properties that emerge at the nanoscale (Yousefi et al. 2023; Mushtaq et al. 2023). Over the past decade, many studies have focused on the development of vitamin D_3_ nanoemulsions using high‐energy methods like ultrasonication and high‐pressure homogenization for developing vitamin D_3_ nanoemulsions, with researchers optimizing ultrasonic parameters for encapsulating vitamin D_3_ in gum arabic (Bashir et al. 2024; Naseema et al. 2021). However, the influence of interfacial composition on the physicochemical stability and bioaccessibility of nanoemulsions remains an evolving field.

Sunflower oil, a predominant culinary oil, is lauded for its health‐promoting attributes. Despite its high‐fat content, it is esteemed for its nutrient composition, particularly its favorable balance of monounsaturated and polyunsaturated fatty acids (Ahmadi et al. 2024; Faraji Sarabmirza et al. 2023; González‐Rámila et al. 2022). These fatty acids are instrumental in modulating cholesterol levels, thereby contributing to cardiovascular health (Alobre et al. 2023). Sunflower oil, with its favorable fatty acid composition, has been studied for its effectiveness in forming stable nanoemulsions for vitamin D_3_ encapsulation (Inapurapu et al. 2023). The increasing consumer preference for sunflower oil is attributable to its health benefits and the growing awareness of dietary impacts on health (Ahmadi et al. 2024; Adeleke and Babalola 2020).

In the realm of food and beverage technology, there is a burgeoning interest in developing advanced colloidal delivery systems. These systems are pivotal for encapsulating lipophilic functional ingredients, such as bioactive lipids, oil‐soluble vitamins, and flavoring agents (Ekrami et al. 2022; Sheybani et al. 2023a; Sheybani et al. 2023b). The encapsulation of these ingredients enhances their stability, bioavailability, and sensory attributes in food products (Hosseini 2021; Sheybani et al. 2023a; Sheybani et al. 2023b). The reduction in particle size to the nanometer range in emulsion‐based systems confers several benefits: increased stability against gravitational separation and droplet coalescence, enhanced optical clarity, and augmented oral bioavailability. These characteristics are particularly beneficial for transparent, aqueous‐based food products, such as beverages and fortified waters (Gohari et al. 2024; Souto et al. 2022).

Vitamin D, existing in two primary forms D_3_ (cholecalciferol) and D_2_ (ergocalciferol), plays a vital role in human health. While vitamin D_2_ is naturally present in certain foods, vitamin D_3_ is synthesized in human skin upon exposure to sunlight (Benedik 2022). However, vitamin D deficiency is prevalent globally, often attributed to inadequate sun exposure, extensive use of sunscreens, or dietary insufficiencies (Grant 2024). Sufficient levels of vitamin D are crucial for preventing a myriad of health issues, including bone diseases, certain cancers, diabetes, and multiple sclerosis. These findings underscore the importance of maintaining optimal vitamin D levels, especially in adult populations (Ao 2021).

Given the challenges in incorporating hydrophobic, sensitive nutrients in food matrices, the food industry is increasingly focusing on fortification strategies, especially for low‐fat foods (Gopi 2022). These nutrients, susceptible to degradation by light, air, and processing, necessitate protective strategies for effective incorporation into food and beverage products (Ekrami et al. 2022). Encapsulation emerges as a viable technique in this context, offering protection to these sensitive compounds and enhancing their solubility in aqueous mediums. Previously, pea protein‐based nanoemulsions have been investigated for their potential to enhance vitamin D_3_ stability and bioavailability, with in vitro studies using Caco‐2 cells demonstrating promising results (Walia and Chen 2020). Recent advancements in the food industry have led to the development of novel, safe encapsulation techniques using natural substances, tailored for the efficient delivery of these nutrients (Ekrami et al. 2022; Rashidi, Faraji Sarabmirza et al. 2022; Rashidi, Nodeh et al. 2022). Notably, oil‐in‐water nanoemulsions are recognized for their efficacy in encapsulating and delivering fat‐soluble vitamins (A, E, D) due to their enhanced digestibility within the human gastrointestinal tract (GIT) (Mu et al. 2023).

The current study aimed to develop a nanoemulsion‐based system for the effective encapsulation of vitamin D_3_ utilizing a high‐energy technique. In contrast to previous studies that have extensively focused on high‐energy methods, our research explores the combined effects of natural stabilizers, such as WPC and pectin, along with Tween 80 as a co‐stabilizer, on the physicochemical stability and bioavailability of the nanoemulsion. While previous work has provided insight into nanoemulsion stability and vitamin D_3_ bioaccessibility (Souto et al. 2022; Thakur 2021). The study comprehensively investigated the optimal conditions for the nanoencapsulation of vitamin D_3_, and evaluated the characterization, stability, and efficacy of these nanoemulsions using advanced analytical techniques such as SEM, DLS, FTIR, and HPLC.

## 2 Materials and Methods

### 2.1 Materials

Vitamin D_3_ (cholecalciferol) was obtained from Sigma‐Aldrich Chemical Company (St. Louis, MO, USA). Sodium azide (NaN_3_) and Tween 80, used as a surfactant, were obtained from the Merck Company (Germany). Pectin, sourced from apple pulp with a high degree of methoxylation (70% esterification), was purchased from the Alifard Company (Iran). WPC, consisting of 35% (w/w) dry protein content, a maximum moisture content of 1.98%, and 3.5% ash, was supplied by the Mihan Dairy Industry Company (Iran). Sunflower oil, used as the oil phase in the nanoemulsions, was acquired from the Sanat Koroush Company. All other chemical reagents were of analytical grade and purchased from Sigma‐Aldrich Chemical Co. (St. Louis, MO, USA). Solutions were prepared using bi‐distilled water.

### 2.2 Methods

#### 2.2.1 Nanoemulsions Preparation

The fabrication of nanoemulsions was executed by adapting a methodology from a previous study, with modifications to optimize the process (Jan et al. 2022). The initial step involved the dispersion of pectin powder in bi‐distilled water, which was continuously stirred at a controlled temperature of 50°C for 1 h. Sodium azide was added at a concentration of 0.02% (w/v) as a preservative to prevent microbial contamination during preparation and storage. This was done by a hydration phase at ambient temperature for 1 day to ensure complete hydration of the pectin molecules. Concurrently, WPC was solubilized in bi‐distilled water and subjected to a 24 h refrigeration period at 4°C, facilitating complete protein hydration.

The amalgamation of the pectin and WPC solutions was conducted under steady stirring conditions at room temperature for 1 h, resulting in a uniform aqueous mixture. The pH of this mixture was adjusted to pH 7.0. This was followed by a heating protocol, where the solution was exposed to 65°C for 30 min. post‐heating, the solution was cooled at 4°C for an additional 24 h, promoting maximal rehydration.

The nanoemulsion formulations, as enumerated in Table 1, were prepared by integrating 10 mg/mL of vitamin‐D_3_ into the oil phase. The oil phase (with vitamin D_3_) was mixed at 800 rpm for 1 h at ambient temperature (25°C) utilizing a magnetic stirrer (MS20D, Witeg, Germany). Subsequently, the oil phase, enriched with vitamin‐D_3_, was incrementally introduced into the aqueous phase, adhering to the proportions delineated in Table 1 while maintaining the agitation speed. The resultant blend underwent a homogenization process employing a high shear homogenizer (WiseTis HG 15D, Germany) at a rotational speed of 15,000 rpm for 10 min duration at ambient temperature (25°C). To mitigate photodegradation of vitamin‐D_3_, all samples were encased in aluminum foil for protection against light exposure.

**TABLE 1:** The composition of the different nanoemulsions.

Sample	WPC (w%)	Pectin (w%)	Water (w%)	Oil (w%)	Surfactant (w%)	Efficiency (%)	Efficiency (%) (Experimental data)
1	1	2	67	27.55	2.5	90.4	85.9
2	2	2	66	27.55	2.5	72.6	69.9
3	2	1	67	27.55	2.5	61.7	58.4
4	1	3	66	27.55	2.5	59.0	55.6
5	1	2	67	2529.5	0.5	63.0	60.0
6	2	2	66	29.525	0.5	50.3	46.5
7	2	1	67	29.525	0.5	48.5	45.5
8	1	3	66	29.525	0.5	44.8	41.2

#### 2.2.2 Nanoemulsions Characterization

##### 2.2.2.1 Particle Size and Zeta Potential Measurements

Dynamic light scattering (Horiba SZ‐100, Japan) was employed to assess the particle size, particle distribution width, and polydispersity index (PDI) of the vitamin‐D_3_‐containing nanoemulsions. This technique allows for the analysis of particle size (range: 0.3 nm to 10 μm), zeta potential (−500 to +500 mV), and molecular weight. The measurements were conducted at a scattering angle of 90°, 1 day post‐nanoemulsion preparation, and periodically over a month. Measurements were carried out at a temperature of 25°C and a pH of 7.4, and the samples were diluted in deionized water at a 1:50 (v/v) ratio. To ensure the accuracy of the particle size and zeta potential data, each measurement was performed in triplicate, and the average values were reported. Zeta potential measurements were performed with an applied electric field of 1 V in the analyzer. Samples were transferred to the capillary tube of the device using a syringe. The span value of particle size distribution was calculated using Formula (1):

(1) $$\text{Span}=\frac{D_{90\%}-D_{10\%}}{D_{50\%}}$$

where *D*_90%_ and *D*_10%_ represent diameters below which 90% and 10% of the particle distribution lies, respectively, and *D*_50%_ is the median diameter.

##### 2.2.2.2 FT‐IR Spectroscopy

FTIR spectroscopy (Bruker Tensor 27, USA) was utilized to identify functional groups, molecular bands, and structural compositions within the nanoemulsions. For this analysis, 2 mg of the nanoemulsion sample was mixed with 500 mg of potassium bromide powder and compressed into tablets. The FTIR spectra were recorded over a wavelength range of 400–4000 nm (Nair et al. 2022).

##### 2.2.2.3 Morphological Analysis

The surface morphology of the nanoemulsions was examined using scanning electron microscopy (SEM, Philips XL ‐ 30). Samples were prepared by depositing a 1 × 1 cm^2^ drop of nanoemulsion on a flat foil surface and subsequently placed on a glass slide. The samples were then dried in a vacuum oven at 30°C for 1 h. The dried samples were coated with a thin gold layer (20% w, 35 nm thickness) using a sputter coater. Imaging was performed at a 20 kV accelerating voltage.

##### 2.2.2.4 Stability Assessment

Nanoemulsion stability was evaluated by storing 15 mL samples in test tubes at 4°C for 60 days. The decision to store the nanoemulsions at 4°C was based on the need to simulate typical refrigeration conditions, commonly used in food storage, which helps maintain the stability and shelf life of emulsions in real‐world applications. Samples were stored in dark conditions to prevent photodegradation of vitamin D_3_ and minimize light‐induced destabilization. All samples were covered with aluminum foil to exclude light exposure. The temperature was maintained using a refrigerated incubator, which ensured consistent cooling at 4°C throughout the storage period. The storage in dark and low‐temperature conditions helped to limit oxidative degradation processes. The creaming percentage, indicative of physical stability, was calculated at intervals of 1, 30, and 60 days using Equation (2):

(2) $$\text{Creaming}\;\left(\%\right)=\frac{V_{t}-V_{s}}{V_{t}}\times 100$$

where *V*_*t*_ is the total volume of the sample, and *V*_*s*_ is the volume of the lower phase layer.

##### 2.2.2.5 Encapsulation Efficiency

The encapsulation efficiency of vitamin‐D_3_ within the nanoemulsions was determined following the method described by Maurya and Aggarwal (2019). Briefly, 1 mL of the nanoemulsion was placed in a centrifugal concentrator tube with a 10 kDa cutoff (Genetix, India) and centrifuged at 15000 rpm for 8 min at 25°C. The supernatant containing free vitamin‐D_3_ was subsequently analyzed by HPLC (Yung Lin 9100, Korea). The encapsulation efficiency (EE) was calculated using the formula (3):

(3) $$\mathrm{EE}\;\left(\%\right)=\frac{\text{amount of encapsulated vitamin}\;D_{3}}{\text{total vitamin}\;D_{3}}\times 100$$

#### 2.2.3 Preparation of Fortified Sunflower Oil

According to guidelines established by the World Food Program (WFP), it is advised that refined sunflower oil be fortified with Vitamin D at concentrations ranging between 2400 to 3600 IU/kg of oil (WF program n.d.). Both vitamin‐D_3_‐spiked and vitamin‐D_3_ encapsulated in nanoemulsions were used for fortification. For thermal stability testing, 10 g samples of vitamin‐D_3_‐spiked sunflower oil and nanoemulsions were heated at 70°C and 130°C for 1 h. A control sample without vitamin‐D_3_ was also included. Subsequently, samples were collected from each treatment for vitamin‐D_3_ residual analysis and stored at 20°C.

#### 2.2.4 Vitamin‐D_3_ Analysis

HPLC (Young Lin 9100, Korea) equipped with a UV detector and a C_18_ reverse‐phase column (particle size 5 μm, diameter 4 mm, length 25 cm) was used to quantify vitamin‐D_3_. The analysis was performed at a wavelength of 265 nm with a mobile phase comprising a 20:80 methanol‐acetonitrile mixture at a flow rate of 1 mL/min. The column temperature was maintained at 45°C. Vitamin concentrations were deduced by comparing with standard chromatograms, estimating the loaded vitamin‐D_3_ concentration in mg/mL.

Vitamin‐D_3_ extraction involved adding 0.5 g of oil samples (vitamin‐D_3_‐spiked and nanoemulsion) to a methanol:2‐propanol (20:80) solution, followed by vortexing for 0.5 min. Then, 1 mL of hexane was added and vortexed for another 1 min. post‐centrifugation at 5000 rpm for 1 h, the upper layer was collected in a dark vial for analysis. This layer was then evaporated, and 0.5 mL of the mobile phase (methanol: acetonitrile, 20:80) was added before HPLC injection. A calibration curve for vitamin‐D_3_ was established at various concentrations to quantify the residual vitamin‐D_3_ in the sunflower oil.

#### 2.2.5 Release of Vitamin‐D_3_ ‐Spiked Sunflower Oil During Storage

A concentration of 10 mg/mL vitamin‐D_3_ was prepared and incorporated into the oil phase, and nanoemulsions were synthesized following the procedure delineated in Section 2.2.1. The encapsulation efficiency of vitamin‐D_3_ within these nanoemulsions was quantitatively assessed. To evaluate the release of vitamin‐D_3_ in the sunflower oil over time, samples containing the nanoemulsion were stored and analyzed at designated intervals. Sampling was conducted at 1, 30, and 60 days during a 60‐day storage period. The aim was to monitor the residual levels of vitamin‐D_3_ in the sunflower oil, providing insights into the release profile and stability of the vitamin within the nanoemulsion matrix under storage conditions. The methodology for extracting the residual vitamin‐D_3_ from the sunflower oil, which contained the nanoemulsion, was consistent with the procedure outlined in Section 2.2.3.

#### 2.2.6 Statistical Analysis

The effects of surfactant and concentration, as well as the ratio of wall materials, were investigated using a factorial design in a randomized design framework. Analysis was performed using SAS statistical software (Version 9 for Windows). Data were analyzed using one‐way ANOVA and Duncan's multiple range test at a 5% significance level, facilitated by SPSS 16 software. The ANOVA test determined the statistical significance of each parameter across three replicates at a 95% confidence level.

## 3 Results and Discussion

### 3.1 Formulation Optimization

The formulation of vitamin‐D_3_ nanoemulsions was optimized by employing vitamin‐D_3_ as the core nucleating agent and a biopolymer solution of pectin and WPC to form the outer aqueous phase wall of the emulsion. The encapsulation process was enhanced through modifications to the biopolymer solution, leveraging the unique properties of pectin to form a three‐dimensional network with polygalacturonic acid chains, which effectively inhibits excessive water absorption (Gaikwad 2024).

A high shear homogenization technique, specifically the emulsion phase inversion (EPI) method, was utilized for nanoemulsion preparation. The encapsulation efficiency across various formulations ranged from 44.8% to 90.4%, as determined using Design Expert software. A detailed analysis using data modeling revealed that encapsulation efficiency correlates directly with the oil encapsulation ratio (Table 1).

The experimental results demonstrated the highest encapsulation efficiency (85.9%) for a formulation with a WPC to pectin ratio of 1:2 and a surfactant concentration of 25% (w/w). Other formulations yielded encapsulation efficiencies as follows: 69.9% (Code 2), 58.4% (Code 3), 55.6% (Code 4), 60.0% (Code 5), 46.5% (Code 6), 45.5% (Code 7), and 41.2% (Code 8), indicating that the empirical data closely align with the predictions made using Design Expert Software. The samples corresponding to each code are illustrated in Figure 3c,d, providing a visual representation and sample composition across formulations.

Further analysis identified the optimal formulation (Code 1) for nanoemulsion fabrication, achieving a 90.4% encapsulation efficiency with the specified WPC:pectin ratio and surfactant concentration. The encapsulation efficiency's variance analysis was conducted using ANOVA, with results presented in Table 2. The model's validity was confirmed with a *p*‐value less than 0.01, indicating a 99% confidence level in the model's predictive capability. Of the two significant models, the second‐grade model was selected for its higher *R* ^2^ value and lower standard deviation, emphasizing its suitability for characterizing encapsulation efficiency.

**TABLE 2:** Analysis of variance to select the formulation for encapsulation efficiency.

Sources of variation	Degree of freedom	Mean square	*F*	*p*	*R* ^2^	Standard deviation
Linear model	4	309.85	2.82	0.0564	0.3688	10.49
2FI model	6	225.49	4	0.0162	0.7594	7.53
2nd order model	4	193.44	42.1	< 0.0001	0.9687	2.13
3rd order model	5	6.19	2.12	0.2089	0.9775	1.72

ANOVA results, with *p*‐values less than 0.05 at a 95% confidence level, underscore the model's significant influence on nanoencapsulation efficiency, reaffirming the critical role of formulation composition in optimizing vitamin‐D_3_ nanoemulsion preparation.

### 3.2 Influence of Aqueous Phase Ratio on Particle Size and Stability of Nanoemulsions

The impact of varying ratios of WPC and pectin on the particle size and distribution within nanoemulsions was systematically investigated. This study specifically evaluated the effects of two distinct ratios of these biopolymers in combination with two concentrations of surfactant (2.5% and 0.5%) on nanoemulsion characteristics, as outlined in Table 3. A notable observation was that an increase in pectin concentration led to a decrease in nanoemulsion particle size, with the smallest particle size recorded at 98.2 nm for the formulation containing 1% w/w WPC and 2% w/w pectin. This trend suggests that pectin plays a crucial role in refining the emulsion matrix to achieve smaller droplets.

**TABLE 3:** Comparison of particle size and span.

Wall composition (%)			Emulsifier (%)		*Z*‐average (nm)	Span
Water	Pectin	WPC	Oil	Surfactant		
67	2	1	527.5	2.5	98.2 ± 0.398.2	0.95
66	3	1	27.55	2.5	84.1 ± 1.184.1	0.83
67	2	1	2529.5	0.5	80.2 ± 0.980.2	0.79
66	3	1	29.525	0.5	75.5 ± 1.075.5	0.72

Additionally, it was observed that a lower concentration of Tween 80 resulted in a narrower particle size distribution, indicating a more uniform dispersion of particles within the nanoemulsion. This effect underscores the significance of surfactant concentration in modulating nanoemulsion homogeneity and stability. Further analysis of the independent variables, namely the core material and wall biopolymer composition, on the nanoemulsion particle size demonstrated statistically significant variations (*p* > 0.05) at the 5% significance level. These variations highlight the sensitivity of nanoemulsion characteristics to the compositional ratios of the constituent materials.

The long‐term stability assessment of the nanoemulsions, focusing on particle size and distribution metrics (Section 3.3), confirmed the robustness of the system. The consistent performance of these parameters over time signifies the effective stabilization of the nanoemulsion structure by the chosen biopolymer and surfactant formulations. Such stability is indicative of the potential applicability of these nanoemulsions in various industrial and pharmaceutical contexts, where long‐term dispersion stability is critical.

### 3.3 Nanoemulsion Characteristic During Storage

The stability of nanoemulsions was assessed under controlled storage conditions at 4°C, which represents typical refrigeration used for food preservation. Samples were stored in dark conditions (covered with aluminum foil) to prevent light‐induced oxidation and degradation of vitamin D_3_. While oxygen was not fully excluded, tight sealing of the vials minimized oxygen exposure. Stability is a crucial parameter for their long‐term efficacy in applications such as encapsulating oils and flavoring materials and has been extensively studied through various parameters including particle size, span index, zeta potential, and the creaming index. These nanoemulsions were characterized by an average particle size of 98.2 nm and formulated with 1% WPC, 2% pectin, and 2.5% surfactant, over a storage period of 60 days, underscoring the effectiveness of the employed fabrication technique in generating stable colloidal systems suitable for long‐term dispersion stability.

**FIGURE 1:**
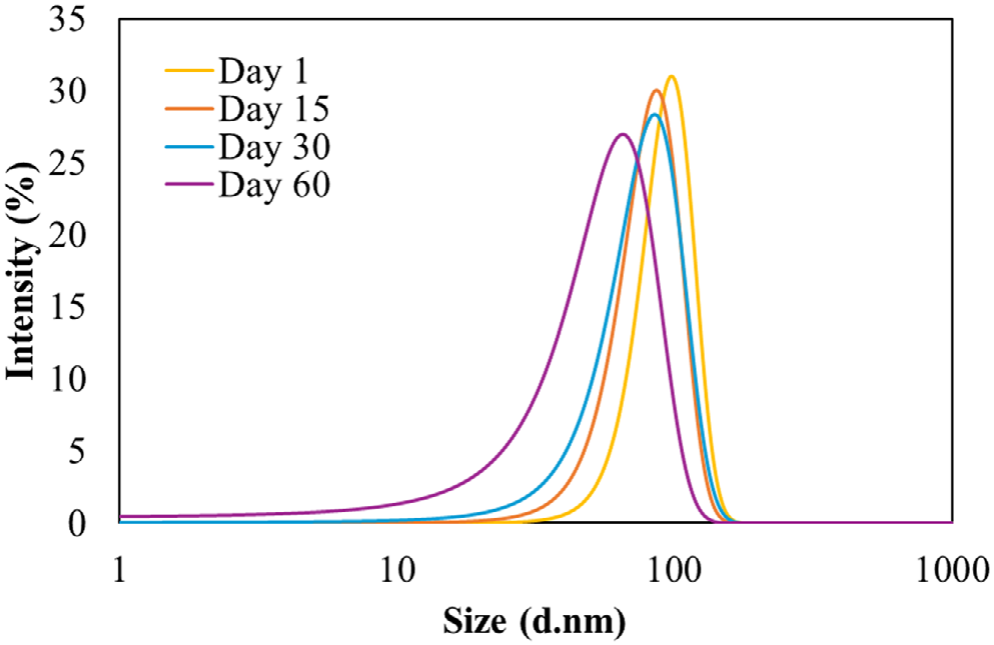
Particle size changes during days 1, 15, 30, and 60 (at 4°C).

DLS analysis was employed to monitor changes in average particle diameter and span index at predetermined intervals (1st, 15th, 30th, and 60th days) post‐nanoemulsion preparation, under constant temperature conditions (Figure 1). As documented in Table 4, the vitamin‐D3 nanoemulsions exhibited remarkable long‐term stability, with no observable phase separation or visual changes throughout the 60‐day evaluation period. Notably, the span index, which indicates the width of the particle size distribution, increased significantly after 15 days, stabilizing after 30 days. This initial increase is attributed to the formation of surface‐activated free micelles, which contribute to a broadening of the particle size distribution during the early stages of storage (15–30 days) due to aggregation phenomena. Beyond 30 days, a reduction in the span index was observed, suggesting a stabilization and integration of particles. Over time, the PDI increased slightly from 0.426 ± 0.05 on day 1 to 0.477 ± 0.05 on day 60, indicating a gradual broadening of the particle size distribution, which could be attributed to emulsion destabilization processes such as coalescence or Ostwald ripening (Guo et al. 2024).

**TABLE 4:** Changes in span index, particle size, Zeta potential, and creaming index during days 1, 15, 30, and 60 (at 4°C).

Day	Particle size (nm)	Span	PDI	Zeta potential (mV)		Creaming index (%)
				Emulsion with vitamin D_3_	Emulsion without vitamin D_3_	
1	98.20 ± 0.3	0.797 ± 0.09	0.426 ± 0.05	−30 ± 0.08	−26 ± 0.04	0
15	86.65 ± 1.3	0.841 ± 0.07	0.430 ± 0.05	−22 ± 0.19	−19 ± 0.41	12.5
30	85.33 ± 2.0	0.918 ± 0.06	0.472 ± 0.05	−20 ± 0.08	−16 ± 0.32	28
60	70.48 ± 3.1	0.912 ± 0.04	0.477 ± 0.05	−19 ± 0.10	−15 ± 0.13	62

A significant observation was the decrease in average particle diameter from 85.33 ± 2.06 nm to 70.48 ± 3.12 nm between the 30th and 60th days of storage, as shown in Table 4. This reduction in particle size, alongside a more homogeneous colloidal solution, underscores the efficiency of the lower surfactant concentration in achieving a narrower particle size distribution. The presence of particles smaller than one micron after 60 days underscores the colloidal system high physical stability and suggests successful mitigation against the Ostwald ripening phenomenon, which is further evidenced by the finer span index (Thakur 2021).

The findings affirm that the employed fabrication technique effectively generates stable nanoemulsions with a homogeneous particle size distribution, crucial for maintaining the physical stability of colloidal systems over time. The observed stability enhancements, particularly in terms of particle size reduction and distribution narrowing, highlight the potential of this formulation for applications requiring long‐term dispersion stability (Culas 2023).

Furthermore, at a standard testing temperature of 25°C, WPC exhibits a negative zeta potential (Table 4). Similarly, pectin molecules also display negative zeta potential values, attributable to variations in their polysaccharide chain structure. The synergistic effect of combining pectin and WPC, particularly through their covalent interactions, was observed to augment the negative charge of the nanoemulsions. This increase in negative surface charge can be attributed to the blocking of lysine's free amino groups and the denaturation of proteins, which alters their spatial configuration, enhancing the emulsion's repulsive forces and thereby its stability (Su et al. 2021). Notably, all nanoemulsions maintained a zeta potential greater than −25 mV, indicative of a high level of colloidal stability. This value could be considered preventative for particle aggregation, as the negative charge promotes repulsion between particles, minimizing the risk of coalescence.

The creaming index study highlights the significance of stability in nanoemulsions, particularly for encapsulating oils and flavoring materials. Creaming serves as a pivotal indicator of instability in oil‐in‐water emulsions, typically leading to phase separation and diminished material preservation capabilities (Ding et al. 2023). Table 4 demonstrates that the creaming index progressively increased with storage time, a trend attributable to hydrophobic interactions and Van der Waals forces, which cumulatively undermine the stability of the emulsion. A significant alteration in the stability index was observed between days 1 and 60 (*p* < 0.05). The observed stability of up to 60 days can be attributed to the formation of fine particles within the emulsion, which counteracts the destabilizing effects of coarse particle aggregation. According to Stokes' law, particle sedimentation velocity is directly proportional to the square of their radius, thus smaller particles contribute to a more stable emulsion (Lorusso et al. 2021). The creaming index provides indirect insights into the particle matter presence within the emulsions over time. The incorporation of WPC was found to enhance stability by creating a physical barrier between droplets and entrapping particles within a three‐dimensional matrix, emphasizing the importance of protein content and particle size in stabilizing nanoemulsions (Falsafi et al. 2022).

### 3.4 FT‐IR Study

**FIGURE 2:**
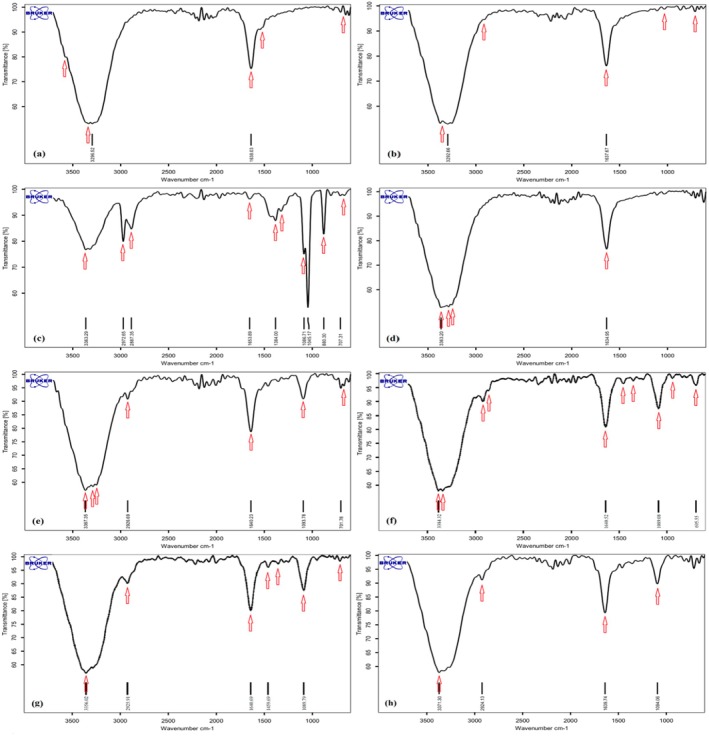
FT‐IR spectra corresponding to (a) WPC, (b) pectin, (c) vitamin‐D_3_ (d) aqueous phase, (e) nanoemulsion, (f) vitamin‐D_3_‐loaded nanoemulsion (on day 1), (g) vitamin‐D_3_‐loaded nanoemulsion after 1 month of storage, and (h) vitamin‐D_3_‐loaded nanoemulsion after 2 months of storage.

The FT‐IR analysis was conducted to elucidate the molecular interactions within the nanoemulsion formulations. The spectra for WPC, pectin, vitamin‐D_3_, an aqueous phase containing a mixture of WPC and pectin, nanoemulsions without vitamin‐D_3_, and nanoemulsions with vitamin‐D_3_ at different stages of storage (day 1, 1 and 2 months) are shown in Figure 2.

The WPC spectrum (Figure 2a) exhibited characteristic bands at 3296 cm^−1^, 1638 cm^−1^, and 1535 cm^−1^, corresponding to the N‐H group stretching, C=O group stretching (amide I band), and C=O stretching (amide II band), respectively (Lou et al. 2023).

Pectin's spectrum (Figure 2b) revealed peaks from 3463 to 3000 cm^−1^ attributed to OH groups, with specific peaks at 1637 cm^−1^ and 1585 cm^−1^ (a weaker peak) corresponding to the stretching C=O of carboxylic acid groups. Additionally, peaks between 1045 and 1080 cm^−1^ were observed, indicating the saccharide structure's C‐O‐C stretching (ether group).

The spectrum for vitamin‐D_3_ (Figure 2c) showed C‐H group tensile vibration peaks at 2887 and 2972 cm^−1^, a weak peak at 707 cm^−1^ for CH_2_ group vibrations, and a peak at 880 cm^−1^ related to C‐H stretching. A prominent peak at 1045 cm^−1^ was attributed to C=C stretching.

The aqueous phase containing a WPC:pectin ratio of 1:2 (Figure 2d) displayed an absence of specific peaks observed individually in WPC and pectin, suggesting a blend effect. The nanoemulsion without vitamin‐D_3_ (Figure 2e) exhibited additional peaks at 2926 cm^−1^, 1083 cm^−1^, and within the 701–770 cm^−1^ range, indicative of C‐H group tensile vibrations, C‐O stretching, and C‐H bending, respectively. These findings suggest predominance of electrostatic forces over chemical interactions in the nanoemulsion matrix. Upon encapsulating vitamin‐D_3_ within the nanoemulsions (Figure 2f), a noticeable shift and reduction in peak intensities were observed, particularly at 2972 cm^−1^ and 2887 cm^−1^, denoting C‐H bond presence. Post vitamin‐D_3_ loading, increased peak intensity at 1089 cm^−1^ for C‐O stretching, along with distinct peaks at 1384 cm^−1^ and 1465 cm^−1^ for C‐H bending, was indicative of vitamin‐D_3_'s integration into the biopolymer network through electrostatic forces. Over one and 2 months of storage (Figure 2g,h), a slight decrease in the intensity of peaks at 1384 cm^−1^ and 1465 cm^−1^ was noted, affirming the stability of vitamin‐D_3_ within the nanoemulsions. This analysis substantiates the electrostatic‐based encapsulation of vitamin‐D_3_, preserving the bioactive compound's integrity and highlighting the nanoemulsion system's capability to maintain stable bioactive encapsulation over time.

### 3.5 Morphological Study

**FIGURE 3:**
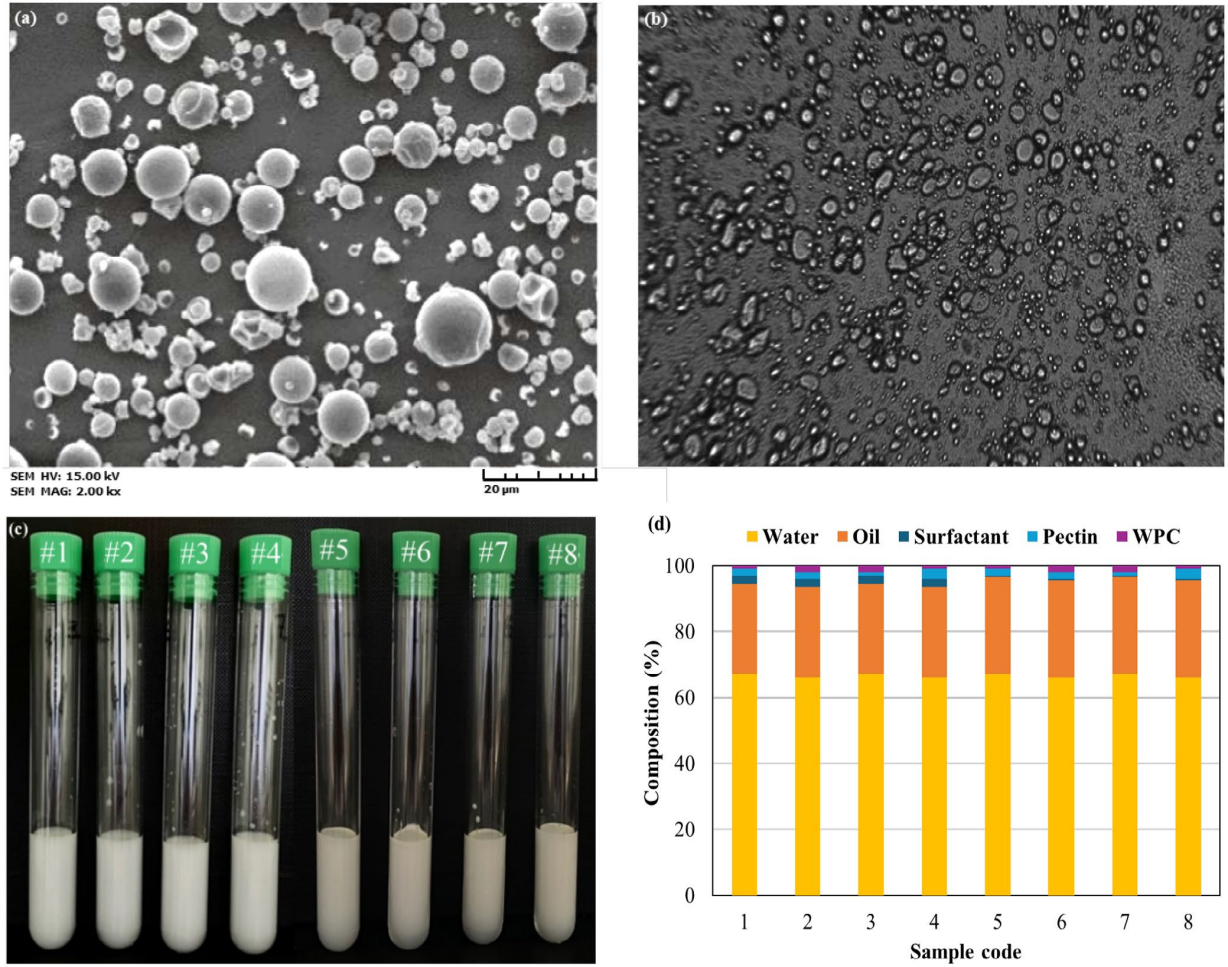
(a) SEM, (b) optical microscopy images, (c) visual inspection, and (d) sample composition of vitamin D_3_ nanoemulsion.

SEM provided a detailed morphological analysis of the nanocoated samples, revealing particle sizes ranging from 100 nm to 10 μm. The majority of the nanoemulsions exhibited spherical morphologies, with a core encapsulated by a smooth nanocoated wall, as depicted in Figure 3a. Additionally, Figure 3b demonstrates the same spherical morphology observed using optical microscopy, further confirming the uniform structure of the nanoemulsions. This morphology underscores the efficacy of the high‐energy method employed for vitamin‐D_3_ nanocoating. A critical observation was the influence of the wall‐to‐core ratio on the nanocoated surface size, with an increase in this ratio leading to larger particle sizes, a phenomenon consistent with previous findings (Göksel Saraç and Doğan 2021). Additionally, emulsions formulated with a lower concentration of wall components were more stable. This stability is attributed to the enhanced coverage and faster rate of nanoemulsion wall formation around the vitamin‐D_3_ core, facilitating the generation of uniformly smooth, planar, and spherical particles.

The flexibility of pectin compared to WPC played a pivotal role in achieving these smooth and spherical surfaces. The combination of pectin and WPC resulted in a homogeneous particle size distribution and cohesiveness within the emulsions, owing to WPC's superior emulsifying properties (Shuai et al. 2022). Furthermore, the formulation's wall composition, nanocoating conditions, and homogenization rate were found to significantly influence the final shape of the emulsion, contributing to the overall stability of the nanoemulsions and their encapsulated compounds (Mushtaq et al. 2023). Although recent research has also explored low‐energy approaches combining pea protein and Tween 80 for efficient vitamin D_3_ delivery (Walia et al. 2022), the observed spherical and planar morphologies of the nanoemulsions underscore the suitability of the high‐energy approach for the effective nanocoating of vitamin‐D_3_, as demonstrated in Figure 3a. This methodology not only ensures the physical stability of the nanoemulsions but also promotes the retention of the bioactive properties of vitamin‐D_3_, making it a promising technique for enhancing the bioavailability of nutraceuticals in food and pharmaceutical applications.

### 3.6 Vitamin‐D_3_ Concentrations and Encapsulation Efficiency

**FIGURE 4:**
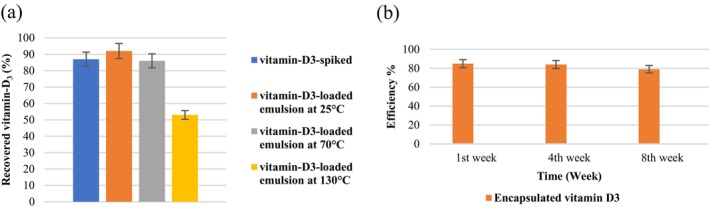
(a) Comparison of mean vitamin D content measured by HPLC for vitamin‐D_3_‐spiked sunflower oil, and vitamin‐D_3_‐loaded emulsion sunflower oil in different temperatures, (b) the efficiency of encapsulated vitamin‐D_3_ during storage.

HPLC was employed to quantitatively analyze the concentration of vitamin‐D_3_ in four distinct samples: vitamin‐D_3_‐spiked sunflower oil, and sunflower oil containing vitamin‐D_3_‐loaded nanoemulsions at 3 different temperature conditions (25°C, 70°C, 130°C). The establishment of a standard curve for vitamin‐D_3_ concentration facilitated the calculation of the area under each chromatographic peak. Chromatogram analyses corresponding to each sample are illustrated in Figure 4a.

Recently, the use of protein‐polysaccharide complexes, such as ovalbumin‐pectin nanocomplexes, has been shown to enhance the storage stability and controlled release of vitamin D_3_ in simulated gastrointestinal conditions (Xiang et al. 2020). The stability of encapsulated vitamin‐D_3_ was systematically assessed over a period of 1, 30, and 60 days, with results presented in Figure 4b. Vitamin‐D_3_'s lipophilic nature permits its encapsulation within a wall material designed to shield against oxidation and hydrolytic degradation. The encapsulation process ensures minimal diffusion of the active component out of the wall structure, thereby enhancing its stability (Pasarin et al. 2023). Factors such as non‐exposure to light and storage in dark conditions were found to contribute significantly to the preservation of encapsulated vitamin‐D_3_. Light and oxygen exposure are known to increase membrane permeability, potentially accelerating the release of vitamin‐D_3_‐loaded emulsion (Talebi et al. 2021).

The choice of oil phase plays a critical role in vitamin D_3_ bioaccessibility, with studies showing that indigestible oil phases, can impact the delivery and absorption of vitamin D_3_ encapsulated in whey protein‐based nanoemulsions (Tan et al. 2019). The efficiency rate, defined as the proportion of the oil phase retained by the capsule wall over time, was evaluated after 2 months of storage, revealing the influence of wall material composition on this parameter. As indicated in Figure 4b, the initial weeks (1–4) showed no significant changes in encapsulation efficiency. However, notable differences emerged after 8 weeks, highlighting the temporal dynamics of capsule performance. Figure 4a showcases the recovery rate of vitamin‐D_3_ in sunflower oil containing vitamin‐D_3_‐loaded emulsion at 92%, compared to 87% in sunflower oil spiked with vitamin‐D_3_. Upon exposure to a temperature of 130°C, a reduction in the recovery rate of vitamin‐D_3_‐loaded emulsion was observed. Nonetheless, the recovery percentage of the encapsulated sample remained relatively stable between ambient temperature and 70°C, underscoring the effectiveness of encapsulation in maintaining vitamin‐D_3_'s integrity under varying thermal conditions.

### 3.7 Temperature Effects on Vitamin‐D_3_ Stability in Sunflower Oil

**FIGURE 5:**
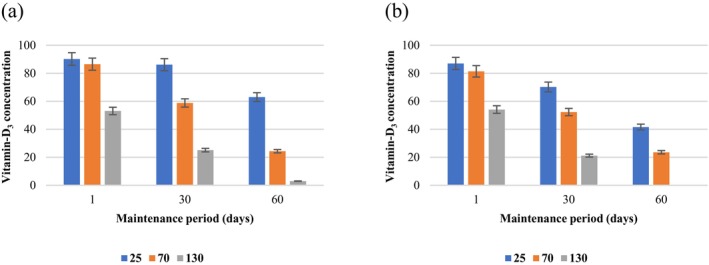
Alterations in vitamin‐D_3_ concentration over time in (a) emulsion samples, (b) spiked samples subjected to storage at 25°C, 70°C, and 130°C.

An analytical observation, as depicted in Figure 5a,b, revealed a behavior between vitamin‐D_3_‐enriched sunflower oil samples with and without encapsulation. The vitamin‐D_3_‐spiked sample exhibited a decreasing concentration trend over time, attributed to the inherent vulnerability of vitamin‐D_3_ to environmental factors such as oxygen, light, and moisture. This degradation is further exacerbated by processes such as saponification and exposure to high temperatures, which can lead to the isomerization and conversion of vitamin‐D_3_ into its precursor forms during soap production (Temova Rakuša et al. 2021).

In contrast, the vitamin‐D_3_‐loaded emulsion sample showed a more stable profile, with a slow‐release pattern trend in vitamin‐D_3_ concentration. The encapsulation effectively forms a protective barrier around the vitamin, significantly mitigating the impact of external factors, especially oxygen exposure. Over time, the encapsulating materials, including pectin, may undergo biochemical reactions such as the Maillard reaction with amino groups in proteins, forming cross‐links that reinforce the barrier against oxygen, thereby preserving the vitamin's integrity (Koczoń et al. 2022).

Quantitative analysis at ambient temperature, 70°C, and 130°C showed no significant differences in the concentrations of both encapsulated and spiked vitamin‐D_3_ initially (Figure 5a,b). However, a difference in vitamin‐D_3_ loss was observed after 60 days of storage, underscoring the effectiveness of encapsulation in protecting vitamin‐D_3_ from degradation under various thermal conditions. This emulsion not only shields the vitamin from light and oxygen but also maintains its stability up to a certain extent when subjected to suitable temperatures. The findings suggest that encapsulation serves as an effective strategy for preserving vitamin‐D_3_'s stability, particularly under conditions that would typically accelerate its degradation. However, the significant impact of high temperatures on emulsion stability observed at 130°C indicates the need for alternative encapsulation techniques or stabilization strategies when exposed to temperatures exceeding 70°C.

## 4 Conclusion

This study successfully demonstrated the efficacy of a high‐energy method for vitamin‐D_3_‐loaded emulsions, utilizing a formulation consisting of 1% w/w WPC, 2% w/w pectin, and an oil phase of 30%. The optimal blending of WPC and pectin resulted in high encapsulation efficiency and effective nanocoating, contributing to remarkable physical stability, rapid lipid digestion, and enhanced bioaccessibility of vitamin‐D_3_ in the developed emulsions. A notable observation was the impact of storage time on the emulsions, with an increase in particle size and a concurrent decrease in stability over time. Interestingly, extending the homogenization period did not significantly affect the reduction in particle size. This suggests a threshold beyond which further homogenization yields diminishing returns in terms of particle size reduction.

The study also revealed that the emulsion fabrication process effectively preserved vitamin‐D_3_ and maintained its stability up to 70°C. These findings underscore the suitability of this approach for vitamin‐D_3_ encapsulation, particularly at moderate temperatures. However, for applications involving temperatures above 70°C, alternative techniques may be warranted to ensure stability. Furthermore, it was observed that increasing the proportions of WPC and emulsifier in the formulation led to a reduction in the droplet size of the oil‐in‐water emulsions. The synthesized nanoemulsions, with an average diameter of 98.2 nm, showed improved encapsulation efficiency and reduced vitamin‐D_3_ loss. This underlines the potential of this encapsulation method in enhancing the stability of vitamin‐D_3_, thereby offering significant benefits for its application in food fortification.

## Author Contributions

Nadia Ahmadi performed tests and wrote the manuscript. Ladan Rashdi revised the draft, and she is the corresponding author. Parham Joolaei Ahranjani reviewed and edited the manuscript. Keramatollah Rezaei reviewed and edited the manuscript.

## Conflicts of Interest

The authors declare no conflicts of interest.

## Data Availability

The current study is available from the corresponding author upon reasonable request.

## References

[fsn34677-bib-0001] Adeleke, B. S. , and O. O. Babalola . 2020. “Oilseed Crop Sunflower ( *Helianthus annuus* ) as a Source of Food: Nutritional and Health Benefits.” Food Science & Nutrition 8: 4666–4684. 32994929 10.1002/fsn3.1783 PMC7500752

[fsn34677-bib-0002] Ahmadi, N. , M. Ghavami , L. Rashidi , M. Gharachorloo , and L. Nateghi . 2024. “Effects of Adding Green Tea Extract on the Oxidative Stability and Shelf Life of Sunflower Oil During Storage.” Food Chemistry: X 21: 101168. 10.1016/j.fochx.2024.101168. 38370306 PMC10869276

[fsn34677-bib-0003] Alobre, M. M. , M. M. Abdelrahman , I. A. Alhidary , A. M. Matar , R. S. Aljumaah , and R. A. Alhotan . 2023. “Evaluating the Effect of Using Different Levels of Sunflower Hulls as a Source of Fiber in a Complete Feed on Naemi Ewes' Milk Yield, Composition, and Fatty Acid Profile at 6, 45, and 90 Days Postpartum.” Sustainability 15: 14431.

[fsn34677-bib-0004] Ao, T. , J. Kikuta , and M. Ishii . 2021. “The Effects of Vitamin D on Immune System and Inflammatory Diseases.” Biomolecules 11: 1624. 34827621 10.3390/biom11111624 PMC8615708

[fsn34677-bib-0005] Bashir, I. , S. M. Wani , N. Jan , et al. 2024. “Optimizing Ultrasonic Parameters for Development of Vitamin D_3_‐Loaded Gum Arabic Nanoemulsions–an Approach for Vitamin D_3_ Fortification.” International Journal of Biological Macromolecules 278: 134894. 10.1016/j.ijbiomac.2024.134894. 39168215

[fsn34677-bib-0006] Benedik, E. 2022. “Sources of Vitamin D for Humans.” International Journal for Vitamin and Nutrition Research 92: 118–125. 34658250 10.1024/0300-9831/a000733

[fsn34677-bib-0007] Culas, M. S. , D. G. Popovich , and A. Rashidinejad . 2023. “Recent Advances in Encapsulation Techniques for Cinnamon Bioactive Compounds: A Review on Stability, Effectiveness, and Potential Applications.” Food Bioscience 57: 103470. 10.1016/j.fbio.2023.103470.

[fsn34677-bib-0008] Ding, B. , S. H. Ahmadi , P. Babak , S. L. Bryant , and A. Kantzas . 2023. “On the Stability of Pickering and Classical Nanoemulsions: Theory and Experiments.” Langmuir 39: 6975–6991. 10.1021/acs.langmuir.3c00133. 37083472

[fsn34677-bib-0009] Ekrami, M. , A. Ekrami , R. H. Moghadam , P. Joolaei‐Ahranjani , and Z. Emam‐Djomeh . 2022. “Food‐Based Polymers for Encapsulation and Delivery of Bioactive Compounds.” in: Biopolymers in Nutraceuticals and Functional Foods. eds S. Gopi , P. Balakrishnan , and M. Bračič Royal Society of Chemistry. 488–544. 10.1039/9781839168048-00488.

[fsn34677-bib-0010] Falsafi, S. R. , A. C. Karaca , L. Deng , et al. 2022. “Insights Into Whey Protein‐Based Carriers for Targeted Delivery and Controlled Release of Bioactive Components.” Food Hydrocolloidal 133: 108002. 10.1016/j.foodhyd.2022.108002.

[fsn34677-bib-0011] Faraji Sarabmirza, R. , P. Joolaei Ahranjani , L. Rashidi , M. Mousavi , F. Khodaiyan , and H. Rashidi Nodeh . 2023. “An Investigation on Conjugated Linoleic Acid Content, Fatty Acid Composition, and Physicochemical Characteristics of Iranian Kurdish Butter Oil.” Food Science & Nutrition 11: 1051–1058. 10.1002/fsn3.3142. 36789035 PMC9922134

[fsn34677-bib-0012] Gaikwad, D. , R. Sutar , and D. Patil . 2024. “Polysaccharide Mediated Nanodrug Delivery: A Review.” International Journal of Biological Macromolecular 261: 129547. 10.1016/j.ijbiomac.2024.129547. 38278399

[fsn34677-bib-0013] Gohari, A. S. , L. Nateghi , L. Rashidi , and S. Berenji . 2024. “Preparation and Characterization of Sodium Caseinate‐Apricot Tree Gum/Gum Arabic Nanocomplex for Encapsulation of Conjugated Linoleic Acid (CLA).” International Journal of Biological Macromolcular 261: 129773. 10.1016/j.ijbiomac.2024.129773. 38296128

[fsn34677-bib-0014] Göksel Saraç, M. , and M. Doğan . 2021. “Encapsulation of Mono,‐Diglycerides Obtained From Rendering Waste Oil: Powder, Interfacial, Rheological and Emulsion Properties.” Journal of Food Processing & Preservation 45: e15520. 10.1111/jfpp.15520.

[fsn34677-bib-0015] González‐Rámila, S. , R. Mateos , J. García‐Cordero , M. A. Seguido , L. Bravo‐Clemente , and B. Sarriá . 2022. “Olive Pomace Oil Versus High Oleic Sunflower Oil and Sunflower Oil: A Comparative Study in Healthy and Cardiovascular Risk Humans.” Food 11: 2186. 10.3390/foods11152186 PMC9331821 35892771

[fsn34677-bib-0016] Gopi, S. , P. Balakrishnan , and M. Brai . 2022. Biopolymers in Nutraceuticals and Functional Foods. London, UK: Royal Society of Chemistry. The Royal Scociety of Chemistry. 10.1039/9781839168048.

[fsn34677-bib-0017] Grant, W. B. , H. P. Bhattoa , and P. Pludowski . 2024. “Determinants of Vitamin D Levels From Sun Exposure: A Global Perspective.” In Feldman and Pike's Vitamin D, 5th edition, 97–113. New York, US: Elsevier. 10.1016/B978-0-323-91338-6.00006-9.

[fsn34677-bib-0018] Guo, Y. , X. Zhang , X. Wang , L. Zhang , Z. Xu , and D. Sun . 2024. “Nanoemulsions Stable Against Ostwald Ripening.” Langmuir 40, no. 2: 1364–1372. 10.1021/acs.langmuir.3c03019. 38175958

[fsn34677-bib-0019] Hosseini, S. F. , L. Ramezanzade , and D. J. McClements . 2021. “Recent Advances in Nanoencapsulation of Hydrophobic Marine Bioactives: Bioavailability, Safety, and Sensory Attributes of Nano‐Fortified Functional Foods.” Trends in Food Science and Technology 109: 322–339. 10.1016/j.tifs.2021.01.045.

[fsn34677-bib-0020] Inapurapu, S. P. , R. Pullakhandam , S. Bodiga , P. S. Yaduvanshi , and V. L. Bodiga . 2023. “Physicochemical Studies of Sunflower Oil Based Vitamin D Nanoemulsions.” Journal of Dispersion Science and Technology 44, no. 8: 1378–1388. 10.1080/01932691.2021.2016440.

[fsn34677-bib-0021] Jan, Y. , L. A. Al‐Keridis , M. Malik , et al. 2022. “Preparation, Modelling, Characterization and Release Profile of Vitamin D_3_ Nanoemulsion.” LWT 169: 113980. 10.1016/j.lwt.2022.113980.

[fsn34677-bib-0022] Koczoń, P. , H. Josefsson , S. Michorowska , et al. 2022. “The Influence of the Structure of Selected Polymers on Their Properties and Food‐Related Applications.” Polymers (Basel) 14: 1962. 35631843 10.3390/polym14101962 PMC9146511

[fsn34677-bib-0023] Lorusso, V. , D. Orsi , F. Salerni , et al. 2021. “Recent Developments in Emulsion Characterization: Diffusing Wave Spectroscopy Beyond Average Values.” Advances in Colloid and Interface Science 288: 102341. 10.1016/j.cis.2020.102341. 33359963

[fsn34677-bib-0024] Lou, C. , X. Liu , C. Yang , F. Ye , and Q. Zhou . 2023. “One‐Step Synthesis of Silver Nanoparticles Exposed on the Chitosan‐Covered Polyamide 6 Electrospinning Nanofibers.” Journal of Applied Polymer Science 140: e53501. 10.1002/app.53501.

[fsn34677-bib-1001] Maurya, V. K. , and M. Aggarwal . 2019. “Fabrication of Nano‐Structured Lipid Carrier for Encapsulation of Vitamin D3 for Fortification of ‘Lassi’; a Milk Based Beverage.” Journal of Steroid Biochemistry and Molecular Biology 193: 105429. 10.1016/j.jsbmb.2019.105429. 31325498

[fsn34677-bib-0025] Mu, H. , Q. Sun , S. Xue , et al. 2023. “Emulsion‐Based Formulations for Delivery of Vitamin E: Fabrication, Characterization, In Vitro Release, Bioaccessibility and Bioavailability.” Food Reviews International 39: 3283–3300. 10.1080/87559129.2021.2011911.

[fsn34677-bib-0026] Mushtaq, A. , S. M. Wani , A. R. Malik , et al. 2023. “Recent Insights Into Nanoemulsions: Their Preparation, Properties and Applications.” Food Chemistry: X 18: 100684. 10.1016/j.fochx.2023.100684. 37131847 PMC10149285

[fsn34677-bib-0027] Nair, A. B. , B. Singh , J. Shah , et al. 2022. “Formulation and Evaluation of Self‐Nanoemulsifying Drug Delivery System Derived Tablet Containing Sertraline.” Pharmaceutics 14: 336. 35214068 10.3390/pharmaceutics14020336 PMC8880292

[fsn34677-bib-0028] Naseema, A. , L. Kovooru , A. K. Behera , K. P. P. Kumar , and P. Srivastava . 2021. “A Critical Review of Synthesis Procedures, Applications and Future Potential of Nanoemulsions.” Advances in Colloid and Interface Science 287: 102318. 33242713 10.1016/j.cis.2020.102318

[fsn34677-bib-0029] Pasarin, D. , A.‐I. Ghizdareanu , C. E. Enascuta , et al. 2023. “Coating Materials to Increase the Stability of Liposomes.” Polymers (Basel) 15: 782. 36772080 10.3390/polym15030782 PMC10004256

[fsn34677-bib-0030] Rashidi, L. , R. Faraji Sarabmirza , P. Joolaei Ahranjani , B. Hadi Jume , Z. Gholami , and H. Rashid Nodeh . 2022. “Dispersive Clean‐Up Process Based on a Magnetic Graphene Oxide Nanocomposite for Determination of 2‐Glycerol Monopalmitate in Olive Oil Prior to GC‐FID and GC‐MS Analysis.” Journal of the Science of Food and Agriculture 102: 995–1001. 10.1002/jsfa.11433. 34302362

[fsn34677-bib-0031] Rashidi, L. , H. R. Nodeh , and S. Shahabuddin . 2022. “Determination of Vitamin D3 in the Fortified Sunflower Oil: Comparison of Two Developed Methods.” Food Analytical Methods 15, no. 2: 330–337. 10.1007/s12161-021-02124-y.

[fsn34677-bib-0032] Rolland, M. , N. P. Truong , K. Parkatzidis , et al. 2021. “Shape‐Controlled Nanoparticles From a Low‐Energy Nanoemulsion.” Journal of the American Chemical Society 1, no. 11: 1975–1986. 10.1021/jacsau.1c00321. PMC8611665 34841413

[fsn34677-bib-0033] Sheybani, F. , L. Rashidi , L. Nateghi , M. Yousefpour , and S. K. Mahdavi . 2023a. “Application of Nanostructured Lipid Carriers Containing α‐Tocopherol for Oxidative Stability Enhancement of Camelina Oil.” Industrial Crops and Products 202: 117007. 10.1016/j.indcrop.2023.117007.

[fsn34677-bib-0034] Sheybani, F. , L. Rashidi , L. Nateghi , M. Yousefpour , and S. K. Mahdavi . 2023b. “Development of Ascorbyl Palmitate‐Loaded Nanostructured Lipid Carriers (NLCs) to Increase the Stability of Camelina Oil.” Food Bioscience 53: 102735. 10.1016/j.fbio.2023.102735.

[fsn34677-bib-0035] Shuai, X. , J. Chen , Q. Liu , et al. 2022. “The Effects of Pectin Structure on Emulsifying, Rheological, and in Vitro Digestion Properties of Emulsion.” Food 11: 3444. 10.3390/foods11213444 PMC9658436 36360057

[fsn34677-bib-0036] Souto, E. B. , A. Cano , C. Martins‐Gomes , T. E. Coutinho , A. Zielińska , and A. M. Silva . 2022. “Microemulsions and Nanoemulsions in Skin Drug Delivery.” Bioengineering 9: 158. 35447718 10.3390/bioengineering9040158 PMC9028917

[fsn34677-bib-0037] Su, J. , Q. Guo , S. Yang , et al. 2021. “Electrostatic Deposition of Polysaccharide Onto Soft Protein Colloidal Particles: Enhanced Rigidity and Potential Application as Pickering Emulsifiers.” Food Hydrocolloidal 110: 106147. 10.1016/j.foodhyd.2020.106147.

[fsn34677-bib-0038] Talebi, V. , B. Ghanbarzadeh , H. Hamishehkar , A. Pezeshki , and A. Ostadrahimi . 2021. “Effects of Different Stabilizers on Colloidal Properties and Encapsulation Efficiency of Vitamin D3 Loaded Nano‐Niosomes.” Journal of Drug Delivery Science and Technology 61: 101284.

[fsn34677-bib-0039] Tan, Y. , J. Liu , H. Zhou , J. M. Mundo , and D. J. McClements . 2019. “Impact of an Indigestible Oil Phase (Mineral Oil) on the Bioaccessibility of Vitamin D3 Encapsulated in Whey Protein‐Stabilized Nanoemulsions.” Food Research International 120: 264–274. 10.1016/j.foodres.2019.02.031. 31000239

[fsn34677-bib-0040] Temova Rakuša, Ž. , M. Pišlar , A. Kristl , and R. Roškar . 2021. “Comprehensive Stability Study of Vitamin D3 in Aqueous Solutions and Liquid Commercial Products.” Pharmaceutics 13: 617. 33922975 10.3390/pharmaceutics13050617 PMC8147103

[fsn34677-bib-0041] Thakur, S. , A. K. Dasmahapatra , and D. Bandyopadhyay . 2021. “Functional Liquid Droplets for Analyte Sensing and Energy Harvesting.” Advances in Colloid and Interface Science 294: 102453. 10.1016/j.cis.2021.102453. 34120038

[fsn34677-bib-0042] Walia, N. , and L. Chen . 2020. “Pea Protein Based Vitamin D Nanoemulsions: Fabrication, Stability and In Vitro Study Using Caco‐2 Cells.” Food Chemistry 305: 125475. 10.1016/j.foodchem.2019.125475. 31518841

[fsn34677-bib-0043] Walia, N. , S. Zhang , W. Wismer , and L. Chen . 2022. “A Low Energy Approach to Develop Nanoemulsion by Combining Pea Protein and Tween 80 and Its Application for Vitamin D Delivery.” Food Hydrocolloids for Health 2: 100078. 10.1016/j.fhfh.2022.100078.

[fsn34677-bib-1002] WF program , n. d. https://www.wfp.org/food‐fortification.

[fsn34677-bib-0044] Xiang, C. , J. Gao , H. Ye , et al. 2020. “Development of Ovalbumin‐Pectin Nanocomplexes for Vitamin D3 Encapsulation: Enhanced Storage Stability and Sustained Release in Simulated Gastrointestinal Digestion.” Food Hydrocolloids 106: 105926. 10.1016/j.foodhyd.2020.105926.

[fsn34677-bib-0045] Yousefi, S. , P. Rajaei , L. Nateghi , H. Rashidi Nodeh , and L. Rashidi . 2023. “Encapsulation of Sesamol and Vitamin A Using Alginate and Chitosan‐Coated W/O/W Multiple Emulsions Containing Tween 80 and Span 80.” International Journal of Biological Macromolecules 242: 124766. 10.1016/j.ijbiomac.2023.124766. 37164132

